# Electroacupuncture Mimics Exercise-Induced Changes in Skeletal Muscle Gene Expression in Women With Polycystic Ovary Syndrome

**DOI:** 10.1210/clinem/dgaa165

**Published:** 2020-03-31

**Authors:** Anna Benrick, Nicolas J Pillon, Emma Nilsson, Eva Lindgren, Anna Krook, Charlotte Ling, Elisabet Stener-Victorin

**Affiliations:** 1 Department of Physiology, Institute of Neuroscience and Physiology, Sahlgrenska Academy, University of Gothenburg, Gothenburg, Sweden; 2 School of Health Sciences, University of Skövde, Skövde, Sweden; 3 Department of Physiology and Pharmacology, Karolinska Institute, Stockholm, Sweden; 4 Epigenetics and Diabetes Unit, Department of Clinical Sciences, Lund University Diabetes Centre, Lund University, Scania University Hospital, Malmö, Sweden

**Keywords:** PCOS, transcriptomics, epigenetics

## Abstract

**Context:**

Autonomic nervous system activation mediates the increase in whole-body glucose uptake in response to electroacupuncture but the mechanisms are largely unknown.

**Objective:**

To identify the molecular mechanisms underlying electroacupuncture-induced glucose uptake in skeletal muscle in insulin-resistant overweight/obese women with and without polycystic ovary syndrome (PCOS).

**Design/Participants:**

In a case-control study, skeletal muscle biopsies were collected from 15 women with PCOS and 14 controls before and after electroacupuncture. Gene expression and methylation was analyzed using Illumina BeadChips arrays.

**Results:**

A single bout of electroacupuncture restores metabolic and transcriptional alterations and induces epigenetic changes in skeletal muscle. Transcriptomic analysis revealed 180 unique genes (*q < 0*.05) whose expression was changed by electroacupuncture, with 95% of the changes towards a healthier phenotype. We identified DNA methylation changes at 304 unique sites (*q < 0*.20), and these changes correlated with altered expression of 101 genes (*P* < 0.05). Among the 50 most upregulated genes in response to electroacupuncture, 38% were also upregulated in response to exercise. We identified a subset of genes that were selectively altered by electroacupuncture in women with PCOS. For example, *MSX1* and *SRNX1* were decreased in muscle tissue of women with PCOS and were increased by electroacupuncture and exercise. siRNA-mediated silencing of these 2 genes in cultured myotubes decreased glycogen synthesis, supporting a role for these genes in glucose homeostasis.

**Conclusion:**

Our findings provide evidence that electroacupuncture normalizes gene expression in skeletal muscle in a manner similar to acute exercise. Electroacupuncture might therefore be a useful way of assisting those who have difficulties performing exercise.

Polycystic ovary syndrome (PCOS) is a complex endocrine and metabolic disorder that affects 5% to 17% of women of reproductive age worldwide, and it is associated with decreased fertility and hyperandrogenism (1, 2). The etiology is unclear, but genetic and epigenetic factors might predispose women to PCOS (3, 4). Heritable factors might account for up to 70% of cases of PCOS, but genome-wide association studies have failed to identify specific genes responsible for the development of PCOS (5). Gene expression can be regulated by epigenetic changes such as DNA methylation (6, 7) and/or histone modification (7). Therefore, interactions between genetic and epigenetic alterations likely lead to the disruption of endocrine and metabolic processes in women with PCOS (3, 4). For example, androgen exposure during fetal life leads to epigenetic reprograming, which in adulthood might lead to the development of PCOS (8, 9)

A hallmark of PCOS is hyperinsulinemia, and these women typically exhibit decreased insulin sensitivity and decreased whole-body glucose uptake, which are associated with an increased risk of developing metabolic disturbances, including type 2 diabetes (10, 11). Women with PCOS present altered transcriptome profiles in their adipose tissue and skeletal muscle (12-14), and regular exercise is therefore a first approach to managing both reproductive and metabolic disturbances in overweight and obese women with PCOS (15). Indeed, a single bout of exercise improves whole-body glucose uptake and induces multiple transcriptional and epigenetic changes in skeletal muscle and adipose tissue (7, 16, 17). However, even if exercise is accessible and affordable, adherence is low due to numerous biological and sociological factors such as lack of motivation and compliance.

Acupuncture with low-frequency electrical stimulation (electroacupuncture) activates pathways similar to those activated by exercise, including metabolic adaptation and sympathetic nerve activation (18-22). Electroacupuncture causes muscle contractions and initiates afferent nerve activity in A-delta and C-fibers (18); we have demonstrated that a single bout of electroacupuncture increases whole-body glucose uptake to a similar extent in overweight and obese women both with and without PCOS (22), and induces multiple transcriptional and epigenetic changes in adipose tissue (23). Electroacupuncture resulting in skeletal muscle contractions might therefore represent an alternative or complementary approach to physical activity for the treatment of PCOS. However, the molecular mechanisms underlying electroacupuncture-induced glucose uptake in skeletal muscle are unknown. We hypothesized that transcriptomic and epigenetic alterations present in skeletal muscle from women with PCOS can be restored by electroacupuncture and that these alterations can mediate glucose uptake. Therefore, the aims were to identify the molecular mechanisms underlying electroacupuncture-induced glucose uptake in skeletal muscle in insulin-resistant overweight/obese women with and without polycystic ovary syndrome (PCOS).

## Materials and Methods

### Human study procedure

The cohort consisted of 21 overweight or obese women (body mass index [BMI] 25-35 kg/m^2^) with PCOS and 21 age, weight, and BMI-matched controls as previously described (22). Of these, we successfully collected skeletal muscle biopsies from 15 women with PCOS and from 14 controls. Therefore, baseline data are only given for those included in the gene expression and DNA methylation analyses (Table 1). Subject recruitment, exclusion and inclusion criteria, clinical examination, and biochemical analyses have been previously described (22). The study was conducted in accordance with the Declaration of Helsinki at Sahlgrenska University Hospital and the Sahlgrenska Academy, University of Gothenburg, Sweden, and was approved by the Regional Ethical Review Board of the University of Gothenburg. All participants gave oral and written informed consent before inclusion. The study was registered at ClinicalTrials.gov (NCT01457209) and is reported according to the CONSORT and STRICTA guidelines (24, 25).

**Table 1.** Characteristics of Women With PCOS and Controls Matched for Age, Weight and Body Mass Index. Cohort Has Been Previously Published (22)

Variable	Controls (*n = *14)	PCOS (*n = *15)	*P*
Age (y)	29.7 ± 5.8	30.7 ± 5.5	0.683
**Body composition**			
Body mass index (kg/m^2^)	30.2 ± 3.6	30.9 ± 4.5	0.591
Weight (kg)	85.0 ± 12.2	85.1 ± 14.3	0.949
Height (cm)	167.6 ± 8.45	166.0 ± 8.2	0.747
Waist-to-hip ratio	0.82 ± 0.06	0.85 ± 0.07	0.270
**Clinical reproductive**			
Antral follicle < 9mm (n)	8.07 ± 4.91	22.36 ± 6.72	**<0.000**
Ovarian volume (cm^3^)	4.68 ± 2.47	9.32 ± 6.32	**0.006**
Oligoamenorrhea/Amenorrhea, n (%)	0 (0)	13 (87)	na
Regular, n (%)	14 (100)	2 (13)	na
Ferriman Gallwey score	1.43 ± 1.40	10.60 ± 6.59	**<0.000**
Acne (yes), n (%)	2 (14.3)	7 (46.7)	na
**Endocrine variables**			
Apolipoprotein A1 (ApoA1) (g/l)	1.46 ± 0.18	1.46 ± 0.22	1.000
Apolipoprotein B (ApoB) (g/l)	0.82 ± 0.17	0.85 ± 0.27	0.983
ApoA1/ApoB ratio	0.56 ± 0.12	0.61 ± 0.18	0.591
Cholesterol (mmol/l)	4.34 ± 0.89	4.73 ± 1.40	0.451
Triglycerides (mmol/l)	0.86 ± 0.31	1.15 ± 0.55	0.112
B-Hba1c (mmol/mol)	31.57 ± 2.77	32.00 ± 2.78	0.683
Glucose (mmol/l)	5.13 ± 0.44	4.82 ± 0.34	0.051
Insulin (mU/l)	9.05 ± 3.83	12.81 ± 8.67	0.310
C-peptide (ng/ml)	0.86 ± 0.35	1.17 ± 0.49	0.057
C-peptide index	5.74 ± 2.63	8.16 ± 3.74	0.062
HOMA-IR	2.02 ± 1.02	3.10 ± 2.28	0.234
HOMA-B	124.3 ± 71.3	201.9 ± 156.4	0.186
Glucose disposal rate (mg × kg^-1^ x min^-1^)	7.47 ± 2.98	6.46 ± 3.11	0.270
**Reproductive variables**			
Testosterone (pg/ml)	262.0 ± 70.9	475.3 ± 190.2	**0.000**
17α-hydroxyprogesterone (nmol/l)	3.11 ± 1.93	2.43 ± 1.02	0.505
Luteinizing hormone (LH) (IU/l)	5.59 ± 2.97	8.13 ± 5.72	0.425
Follicle stimulating hormone (FSH) (IU/L)	5.66 ± 3.42	4.09 ± 1.31	0.123
LH/FSH ratio	1.18 ± 0.84	1.95 ± 1.17	0.158
Sex hormone binding globulin (nmol/liter)	45.29 ± 27.45	37.07 ± 15.81	0.505

**Figure 1.**
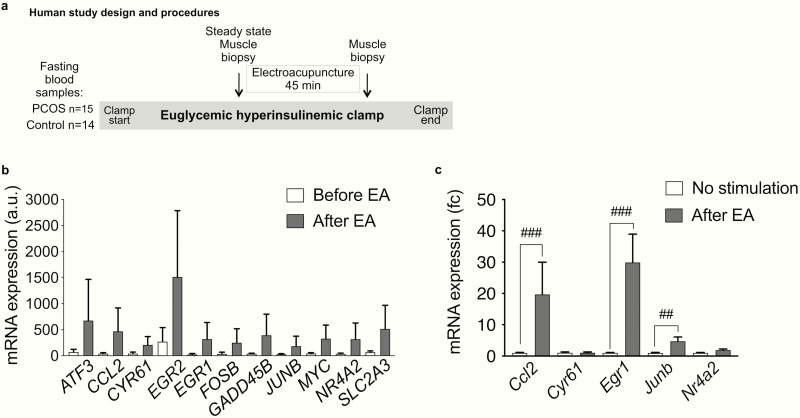
Electroacupuncture induces similar gene expression response in human and rat skeletal muscle**. (a)** Overview of the human study design and procedures: single bout of electroacupuncture in women with and without PCOS. **(b)** The 6 genes with the largest increase in mRNA expression and the 5 selected genes involved in muscle function and metabolism in skeletal muscle in response to a single bout of electroacupuncture (EA) in 15 women with PCOS, based on linear regression analyses (*q < *0.05); data presented as mean ± SD. All differentially expressed genes identified by genome-wide arrays are presented in Supplementary Table S2 (30). **(c)** Skeletal muscle (EDL) gene expression in rat after a single bout of electroacupuncture (n = 9) and nonstimulated controls (n = 5); data presented as mean ± SEM of fold change (fc) and analyzed with Mann-Whitney U test; ^##^*P* < 0.01, ^###^*P* < 0.001.

We used a fixed protocol based on a Western medicine style of acupuncture (26). A single bout of acupuncture with a combination of manual and low-frequency electrical stimulation of the needles, so-called electroacupuncture, causing muscle contractions was given during a euglycemic-hyperinsulinemic clamp. The study design is portrayed in Fig. 1a and the procedure has been previously described (22). In controls, blood samples were obtained in the morning after an overnight fast during the early follicular phase (days 1-10 of the menstrual cycle) in order to match the hormonal milieu of PCOS subjects and to avoid the preovulatory increase in estrogen. Fasting blood samples were collected independently of cycle day in oligo-/anovulatory women with PCOS. In brief, insulin was infused (40 mU/min/kg), and the glucose infusion rate was adjusted to reach a steady state. Blood glucose levels were measured every 5 minutes using a OneTouch Ultra2 device (LifeScan, Inc., Milpitas, CA). At steady state, skeletal muscle biopsies from the vastus lateralis were obtained under local anesthesia (Xylocaine, AstraZeneca AB, Södertälje, Sweden) and snap frozen in liquid nitrogen and stored at −80°C. Immediately after the biopsy, acupuncture needles (40 mm × 0.30 mm, HEGU Svenska AB, Landsbro, Sweden) were inserted to a depth of 15 to 40 mm bilaterally in acupoints in the abdominal muscles (conception vessel [CV] 3 and 12, stomach ([ST] 29), located in the same somatic innervation area as the ovaries and pancreas, and in quadriceps muscles (ST 32 and 34) with the aim to activate large muscles. In addition, needles were placed in muscles below the knee (ST36) and spleen (SP) 6 and in the hand (large intestine 4). When inserted, all needles were stimulated by manual rotation until needle sensation was felt (*de qi*). Needles in the quadriceps and abdominal muscles were attached to electrodes and electrically stimulated at low frequency (2 Hz) (CEFAR ACUS4; Cefar-Complex Scandinavia, Sweden), causing muscle contractions. Needles not connected to the electrical stimulator were stimulated every 10 minutes by manual rotation. Immediately after 45 minutes of electroacupuncture, a second muscle biopsy was obtained.

### Biochemical analyses

Circulating levels of sex hormones were measured by GC-MS/MS as previously described (27), and 17α-hydroxyprogesterone was measured by radio-immunoassay. An accredited laboratory at the Department of Clinical Chemistry of Sahlgrenska University Hospital analyzed insulin, glycated hemoglobin A1c (HbA1c), total cholesterol, triglycerides, apolipoprotein A1, apolipoprotein B, sex hormone–binding globulin, luteinizing hormone, and follicle-stimulating hormone. Serum C-peptide was measured with a human diabetes C-peptide magnetic bead set (Bio-Rad, Hercules, CA, USA).

### Rat study procedure

The animal experiments were approved by the Animal Ethics Committee at the University of Gothenburg, Sweden, and followed the Guide for the Care and Use of Laboratory Animals. Female Wistar rats (Charles River, Frankfurt, Germany) arrived at 13 weeks of age and were fed *ad libitum* with standard chow diet (Harlan Teklad Global Diet, Frankfurt, Germany). Vaginal smears were performed to determine estrus cycle stage by microscopic analysis (28). Rats in estrus phase were selected and subjected to a euglycemic-hyperinsulinemic clamp and randomly divided into the no-stimulation group (n = 5) or the low-frequency electroacupuncture group (n = 9). The euglycemic-hyperinsulinemic clamp was performed in anesthetized rats (Inactin 150 mg/kg, i.p.; Sigma-Aldrich, St. Louis, LA). Catheters were inserted into the right jugular vein for constant infusion of insulin (8 mU ∙ kg^−1^∙min^−1^) and 20% saline glucose solution to maintain a glucose concentration of 6 mmol/L. Blood was drawn from the left carotid artery, and blood glucose levels were measured every 5 minutes (OneTouch Ultra 2, LifeScan, Inc., Milpitas, CA). At steady state, 2 acupuncture needles (0.20 × 15 mm, HEGU Svenska AB, Landsbro, Sweden) were inserted bilaterally in the rectus abdominis muscle (corresponding to acupuncture points ST27-ST29) and 2 were inserted in triceps surae muscles (corresponding to SP6 and SP9). All acupuncture points were in somatic segments corresponding to the same innervation area as the ovaries and pancreas. After insertion, the needles were attached to an electrical stimulator (CEFAR ACU II; Cefar-Complex Scandinavia, Sweden) and were subjected to low-frequency stimulation (2 Hz) causing visible muscle contractions for 45 minutes. After 45 minutes of electroacupuncture, the rats were observed for 60 minutes. Animals in the no-stimulation group underwent the same procedure but without needle insertion. Rats were euthanized by decapitation, and the extensor digitorum longus (EDL) muscle was harvested for molecular analysis.

### In vitro electrical pulse stimulation of myotubes

A cell bank of satellite cells was established from human muscle biopsy samples of the vastus lateralis muscle of healthy male and female volunteers. The biopsies were obtained with informed written consent and approval by the Regional Ethical Review Board of the Karolinska Institute (Stockholm, Sweden). Muscle cells were grown and differentiated as described previously (29). Fully differentiated myotubes grown in 6-well plates were washed with PBS, and 2 mL of postfusion medium containing 5 mM glucose was added per well. Cells were pulsed using the C-Pace EP Culture Pacer (IonOptix) according to 2 different protocols. Acute exposure was 40 V with a 2 ms pulse duration at 1 Hz for 3 hours. Chronic/training exposure was intervals of 40 V with a 2 ms pulse duration at 1 Hz for 30 minutes followed by 3 hours of rest.

### Gene silencing

Six days after inducing differentiation, cells were transfected with 10 nM of either Silencer Select Negative Control No0.2 (no. 4390847) or validated Silencer Select siRNA to target AKAP13 (s680), HMOX1 (s194530), MSX1 (s224066), and SRXN1 (s44409, Life Technologies). The selection of target genes was based on those that were differentially regulated between PCOS and controls at baseline and that were also altered by electroacupuncture. One gene was not detected in myotubes, and 6 genes had a fold change difference < 0.2 between controls and PCOS. Of the 9 remaining genes, we selected the 4 genes with the highest expression in human myotubes (Supplementary Fig. S1 (30). Transfections were performed in OptiMEM reduced-serum media with Lipofectamine RNAiMAX transfection reagent (Invitrogen). Cells were exposed to 2 separate 5-hour transfection periods separated by ~48 hours. Two days after the final transfection, silencing efficiency was estimated using qPCR and insulin-stimulated glucose conversion into glycogen determined as described previously (31).

### RNA extraction and mRNA expression arrays

Skeletal muscle mRNA was extracted from biopsies of 15 women with PCOS and 14 healthy controls and from rat skeletal muscle using the RNeasy Mini Kit (Qiagen). Cultured muscle cells were lysed and RNA was extracted using the E.Z.N.A Total RNA Kit (Omega Bio-tek, Norcross, GA). Nucleic acid concentration and purity were estimated with a NanoDrop spectrophotometer (Thermo Scientific, Wilmington, DE), and RNA quality was determined with an automated electrophoresis station (Experion, Bio-Rad).

To assess the global mRNA expression profile in skeletal muscle, we analyzed isolated mRNA from 15 of the women with PCOS using HumanHT-12 v4 Expression BeadChips (Illumina). cRNA synthesis, including biotin labeling, was carried out using an Illumina TotalPrep RNA Amplification Kit (Life Technologies & Invitrogen) according to the manufacturer’s recommendations. Biotin-cRNA complex was then fragmented and hybridized to the probes on the Illumina BeadChip array. Probes were hybridized and stained with streptavidin-Cy3 before visualization with an Illumina HiScan fluorescence camera. The Oligo package from Bioconductor was used to compute robust multichip average expression measures (32).

For a more targeted approach, custom-designed TaqMan low-density array microfluid cards (Applied Biosystems) were used for quantitative real-time PCR amplification of 44 target genes and 4 reference genes in 14 controls and 15 women with PCOS, as well as in electrical pulse stimulation (EPS)-treated myotubes. The selected target genes were those that were differentially regulated between PCOS and controls at baseline and that were also differentially regulated by both exercise and electroacupuncture. This generated 89 genes; we then filtered out noncoding regions and kept only genes that were expressed in the opposite direction after electroacupuncture compared to the expression in PCOS and controls at baseline, resulting in 46 genes (Supplementary Fig. S1 (30)). The least abundant gene was excluded, and 1 gene was not available as a TaqMan assay (Supplementary Table S1 (30)). cDNA was synthesized from total RNA using the High Capacity RNA-to-cDNA kit (4387406, Applied Biosystems, Carlsbad, CA), and array cards were run on a ViiA 7 Real-Time PCR system thermal cycler according to the manufacturer’s instructions. The NormFinder algorithm (33) was used to calculate the most stable reference genes, and gene expression results were then normalized against the expression of *HPRT1* and *TBP*, which showed the lowest variability in muscle tissue and myotubes.

SYBR Green real-time PCR reactions (Power SYBR Green PCR Master Mix, Applied Biosystem, Hercules, CA, USA) were performed using StepOnePlus Real-Time PCR Systems (Applied Biosystem) for detection and quantification of mRNA in rats to replicate genes with the largest expression difference after electroacupuncture in women with PCOS. Specific pairs of PCR primers for SYBR green detection were also used (Assay ID qRnoCED0009272, qRnoCED0004818, qRnoCED0001041, qRnoCED0002473, qRnoCED0007 596, Bio-Rad, Hercules, CA, USA). The gene expression results were normalized to the expression of *Gapdh* in rats which showed the lowest variability using NormFinder (33). Gene expression values were calculated with the ΔΔ Ct method.

### DNA extraction and DNA methylation arrays

For methylation array studies, DNA was isolated from skeletal muscle biopsies taken before and after a single bout of electroacupuncture from 14 women with PCOS using the QIAamp DNA Mini Kit (Qiagen). Nucleic acid concentrations and purity were estimated with a NanoDrop spectrophotometer (Thermo Scientific, Wilmington, DE), and DNA integrity was checked by gel electrophoresis.

Genome-wide DNA methylation in skeletal muscle from women with PCOS was analyzed with Illumina Infinium HumanMethylation450k array BeadChips. The array contains 485 577 cytosine probes covering 21 231 (99%) RefSeq genes (34). A DNA Methylation Kit (D5001-D5002, Zymo Research) was used to convert genomic DNA to bisulfite-modified DNA. Briefly, gDNA (500 ng) of high quality was fragmented and hybridized on the BeadChip, and the intensities of the signals were measured with a HiScanQ scanner (Illumina).

The bioinformatics analyses were performed as described previously (35). In brief, Y chromosome probes, rs-probes, and probes with an average detection *P* value > 0.01 were removed. After quality control and filtering, methylation data were obtained for 481 089 CpG sites. Beta-values were converted to M-values (M = log2 (β/(1 − β), which were used for all data analyses. Data were then quantile-normalized and batch corrected with COMBAT (36). To improve interpretation, after all the preprocessing steps the data were reconverted to beta-values ranging from 0% (unmethylated) to 100% (completely methylated), and these are presented in the figures.

### Meta-analysis

To investigate similarities in gene expression changes in response to a single bout of exercise compared to the electroacupuncture results presented here, we performed a meta-analysis including 3 studies comprising a single bout of aerobic or resistance exercise in 16 women, aged 23 to 33 years, with a BMI of 24 to 27 kg/m^2^ (*q < *0.05, GSE43219, GSE71972 and GSE28422) (37).

### Pathway analyses

All pathway analyses were performed using R 3.5.2 (www.r-project.org). Transcripts were ranked according to the t-statistics in a *t* test, and we applied gene set enrichment analysis (GSEA) (38) to the expression array data using gene ontology (geneontology.org) and the R package cluster profiler (39) on genes that passed an FDR < 0.05. The GSEA considered pathways with 1 to 500 transcripts.

### Motif enrichment

Motif enrichment analysis was performed to determine which DNA-binding transcription factors might regulate the transcription of each dataset by detecting the enrichment of known binding motifs in the genes’ regulatory regions. Genes were filtered at FDR < 0.05 and ranked by fold-change, and the top 100 increased genes were selected and their promoters were collected from the eukaryotic promoter database (https://epd.vital-it.ch). Regions from −499 to 100 bases were selected in the most representative promoter for each gene. Analysis of motif enrichment was performed using the MEME suite 5.0.5 (http://meme-suite.org) and the HOCOMOCO v11 database.

### Statistical analysis

Differences in clinical characteristics between women with PCOS and controls were based on the Mann–Whitney U-test. Changes in skeletal muscle DNA methylation and gene expression between before and after a single bout of electroacupuncture were based on linear regression analysis. The FDR was used to correct for multiple testing. The chi-squared test was used to calculate whether the altered methylated sites were more than the expected number by chance. Spearman correlation analyses were performed to determine if the electroacupuncture-induced changes in the glucose infusion rate correlated with changes in DNA methylation or gene expression. Human data for DNA methylation and gene expression are presented as the mean ± standard deviation (SD). Gene expression in rat skeletal muscle after electroacupuncture was not normally distributed and was analyzed with the Mann–Whitney U-test. Gene expression in skeletal muscle in PCOS and controls between before and after a single bout of electroacupuncture and in EPS-treated myotubes was analyzed by Student *t* test and presented as the mean ± standard error of the mean (SEM). The effect of insulin and siRNA on myotubes was analyzed by two-way ANOVA. All statistical analyses were performed using the SPSS software (version 24; SPSS, Inc., Chicago, IL).

## Results

### Clinical characteristics

Women with PCOS had more antral follicles, larger ovaries, higher Ferriman–Gallwey scores, and increased levels of circulating androgens than women without PCOS (Table 1). Eleven of the 15 women with PCOS met all 3 Rotterdam criteria. As previously described, a single bout of 45 minutes of low-frequency electroacupuncture increased whole-body glucose uptake in women with and without PCOS as measured by the euglycemic-hyperinsulinemic clamp technique (22).

#### Skeletal muscle gene expression changes after a single bout of electroacupuncture.

We analyzed genome-wide mRNA expression in skeletal muscle from 15 women with PCOS. After correction for multiple testing by false discovery rate (FDR* < *0.05), 180 unique transcripts exhibited changed expression in skeletal muscle after a single bout of electroacupuncture (Supplementary Table S2 (30)). Of these transcripts, 122 were upregulated and 58 were downregulated in women with PCOS, and the expression changes of the transcripts ranged from −43% to +1146%. The 6 genes with the largest expression increases were *EGR2*, *CCL2*, *GADD45B*, *ATF3*, *NR4A2*, and *SLC2A3*, and many of the identified genes are known to play important roles in muscle function and metabolism, including *CYR61*, *EGR1*, *FOSB*, *JUNB*, and *MYC* (Fig. 1a) (40-42). The gene expression of 5 of these genes were also measured in rats by RT-qPCR after a single bout of electroacupuncture. *Ccl2*, *Egr1*, and *Junb* were upregulated in a similar manner in rat skeletal muscle after electroacupuncture, while expression of *Nr4a2* and *Cyr61* was unaltered (Fig. 1b).

#### Genome-wide DNA methylation in skeletal muscle in response to electroacupuncture.

Of the ~481 000 analyzed CpG sites, 60 063 sites (12.5%) had changes in DNA methylation in muscle tissue after a single bout of electroacupuncture (based on *P < *0.05, which is more than expected by chance (*P* < 0.0001, chi-squared test)). However, no sites were significantly changed after FDR correction below 5% (*q* < 0.05), although 304 CpG sites remained at *q < *0.20 and *P* = 10^−4^ (Supplementary Table S3 (30)). The absolute changes in methylation ranged from −7.7% to +3.6% points. The vast majority of these CpG sites (278 sites; 91%) displayed decreased DNA methylation in response to electroacupuncture.

### Overlap between gene expression and DNA methylation

**Figure 2.**
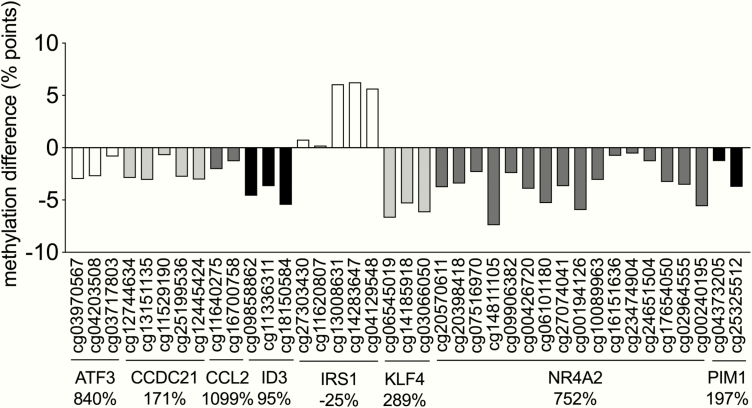
More than 50% of the unique transcripts with a change in expression in response to electroacupuncture had one or several CpG sites annotated to the gene with a change in DNA methylation. CpG sites (*P* < 0.05) with a change in DNA methylation annotated to genes with altered expression (*q < 0*.05). Values are change before *vs* after electroacupuncture presented as % points for CpG site methylation and % change in gene expression presented next to each gene name. All altered CpG methylation sites of genes with changes in mRNA expression in response to electroacupuncture were calculated based on linear regression analyses and are presented in Supplementary Table S4 (30).

In total, 101 of the 180 unique transcripts with a change in expression in response to electroacupuncture had DNA methylation changes at one or several CpG sites (*P* < 0.05; in total 347 CpG sites). Among these CpG sites, the majority (69.5%) had methylation and expression changes going in the opposite direction (Fig. 2 and Supplementary Table S4 (30)), supporting the concept that increased methylation is associated with gene silencing.

### Correlations between changes in glucose infusion rate and mRNA expression

To determine if the electroacupuncture-induced changes in the glucose infusion rate, as measured by a euglycemic-hyperinsulinemic clamp, correlated with electroacupuncture-induced changes in gene expression (Illumina HumanHT-12 BeadChips array), we performed Spearman correlation analyses. Among the genes with a significant expression change in response to electroacupuncture (*q < *0.05), we identified 16 genes that correlated with increased glucose infusion rate, including *KLF4*, *NR4A2*, *ID3*, and *PIM1* (*P* < 0.05). Eight genes also had several CpG sites with decreased methylation annotated to them (Supplementary Table S5 (30)).

### Enrichment of known binding motifs in regulatory regions

**Figure 3.**
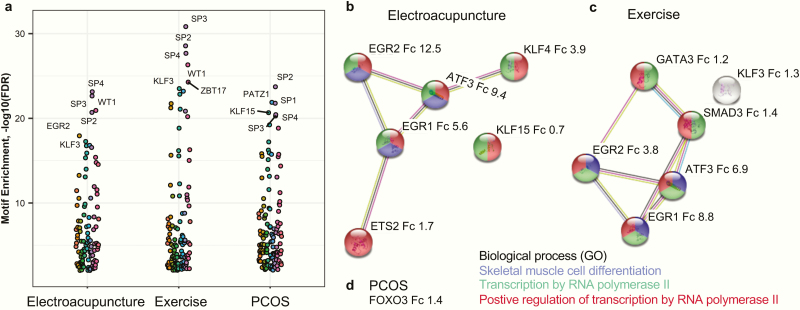
Transcription factors could be direct regulators of gene sets altered in women with PCOS or in response to electroacupuncture or exercise. (a) Motif enrichment analysis of the top 100 genes with the largest fold increase in each condition, and Supplementary Table S6 (30). Fold change (Fc) of transcription factor gene expression in response to (b) electroacupuncture, (c) exercise, and (d) PCOS (*q < *0.05) based on linear regression analyses.

Next we performed a motif-enrichment analysis to determine which DNA-binding transcription factors might be direct regulators of the sets of genes that were altered in women with PCOS and/or in response to electroacupuncture or exercise. The transcription factors SP1-4 act as activators or repressors of gene expression depending on the physiological or pathological stimuli, and these transcription factors were predicted to regulate gene expression in all gene sets (Fig. 3a and Supplementary Table S6 (30)). The transcription factors PATZ1, KLF15, and MAZ are likely to bind to genes that are altered in women with PCOS compared to controls, while WT1, EGR2, and KLF3 are predicted to be involved in the response to exercise and electroacupuncture (Fig. 3a). Six transcription factors had altered gene expression after exercise and electroacupuncture, respectively (Fig. 3b-c), while *FOXO3* was upregulated in women with PCOS vs controls (Fig. 3d). *EGR1*, *EGR2*, and *ATF3* were the transcription factor transcripts with the greatest increases in expression in response to both exercise and electroacupuncture.

### Electroacupuncture and exercise show similar changes in gene expression and signaling pathways

**Figure 4.**
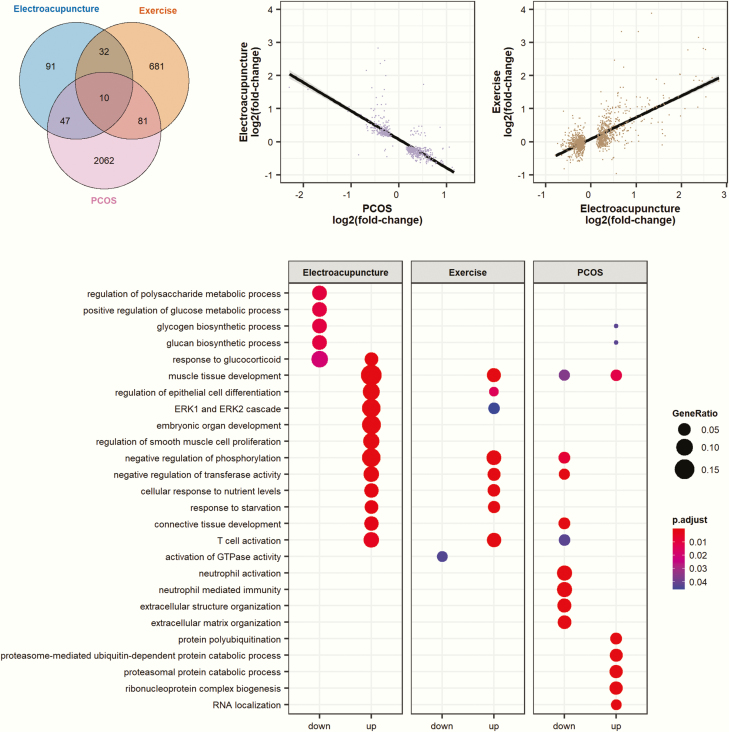
One bout of electroacupuncture or exercise shows similarities in gene expression changes and signaling pathways. Comparison of genes and pathways responding to electroacupuncture, exercise and PCOS. **(a)** Number of genes significantly modified by electroacupuncture (*q < *0.05), exercise (*q < *0.05), and PCOS (*P* < 0.05) were overlapped in a Venn diagram. (**b)** Correlation of fold-changes of genes significantly modified by electroacupuncture (*q < *0.05) with genes changed in women with PCOS. Spearman *r* = -0.79. **(c)** Correlation of fold-changes of genes significantly modified by exercise (meta-analysis (37), *q < *0.05) with genes modified by electroacupuncture in the present study. Spearman *r* = 0.59. **(d)** Overrepresentation analysis using gene ontology on genes significantly modified by electroacupuncture (*q < *0.05), exercise (*q < *0.05) or PCOS (*P* < 0.05).

**Figure 5.**
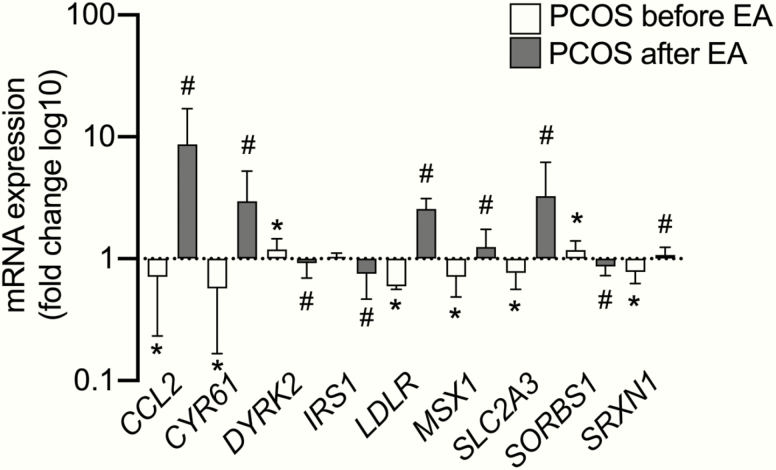
Many genes regulated in response to electroacupuncture changed toward a healthier phenotype. Genes differentially expressed in muscle from PCOS vs controls (controls are set to 1) before electroacupuncture (EA)(*P < 0.05), and genes changing after a single bout of acupuncture (^#^*q* < 0.05). Data presented as fold change mean ± SD and analyzed with Student *t* test. Overlap between all differentially expressed genes are presented in Supplementary Table S7 (30).

We next searched for overlap between genes that significantly changed their expression in response to electroacupuncture and the differentially expressed skeletal muscle genes between women with and without PCOS that were identified in our previously published study (43), all of which were assessed by Illumina HumanHT-12 BeadChips arrays. Of the 2200 differentially expressed genes in skeletal muscle identified in women with PCOS (*P* < 0.05), 57 were regulated in response to electroacupuncture (*q < *0.05, Fig. 4a, Supplementary Table S7 (30)), and for 54 of these genes (95%) the changes were directed toward a healthy phenotype as illustrated for *CCL2*, *CYR61*, *DYRK2*, *IRS1*, *LDLR*, *MSX1*, *SLC2A3*, *SORBS1,* and *SRXN1* (Fig. 5). Next, we identified an inverse correlation for differentially expressed genes in skeletal muscle in women with and without PCOS and genes that changed in response to electroacupuncture (*r*_*s*_ = −0.79, Fig. 4b). According to gene ontology analysis, the most enriched signaling pathways in response to electroacupuncture included those involved in the regulation of glucose metabolism and muscle tissue development (Fig. 4d). Muscle and connective tissue development pathways downregulated in women with PCOS were upregulated by electroacupuncture, while glycogen biosynthetic pathways were downregulated by electroacupuncture. Genes contributing to the enrichment of the top 10 up and downregulated gene sets are shown in Supplementary Table S8 (30).

To determine possible similarities and cross-talk between skeletal muscle and adipose tissue, we searched for overlap between genes whose expression was changed in response to electroacupuncture in muscle in the present study and in adipose tissue from a previous study (23), all of which were assessed by genome-wide expression differences. Out of the 180 genes with altered expression in skeletal muscle in response to electroacupuncture, 114 were also altered in adipose tissue (Supplementary Table S9 (30)), and 100 of these overlapping genes (88%) were regulated in the same direction in both tissues.

To determine any similarities in gene expression changes in response to a single bout of low-frequency electroacupuncture and a single bout of exercise in healthy women, we performed a meta-analysis of 3 studies of a single bout of exercise in women of similar age but with slightly lower BMI (40, 41, 44). Forty-two of the genes that changed expression following exercise in skeletal muscle in these studies (*q < *0.05) also changed expression in response to electroacupuncture in the present study, 34 genes were upregulated and 8 downregulated (Fig. 4a, Supplementary Table S10 (30)). Among the 50 most upregulated genes in response to electroacupuncture, 38% were also upregulated in response to exercise. This was further confirmed by a strong positive correlation between electroacupuncture-induced changes in gene expression (*q < *0.05) in this study and changes in gene expression (*q < *0.05) after 1 bout of exercise (40, 41, 44) (*r*_*s*_ = 0.59, Fig. 4c). Gene ontology analysis showed that 9 out of the 17 enriched signaling pathways overlapped between electroacupuncture and exercise (Fig. 4d).

**Figure 6.**
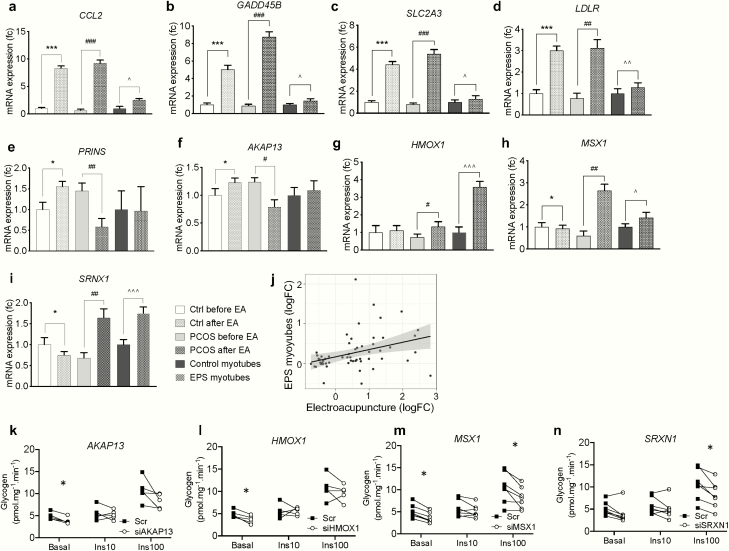
Twenty-five percent of validated genes were upregulated in response to electrical stimulation in women with and without PCOS, and in myotubes following EPS. (a-d) Effect of electrical stimulation on genes with the largest changes in mRNA expression in PCOS, controls and myotubes (targeted approach)*; CCL2, GADD45B, SLC2A3, LDLR*. (e-i) Genes regulated in opposite directions in response to electrical stimulation in PCOS (n = 15) and controls (n = 14); *PRINS, AKAP13, HOMOX1, MSX1, SRNX1.* *P < 0.05, ***P < 0.001 vs control before EA, ^#^*P* < 0.05, ^##^*P* < 0.01, ^###^*P* < 0.001 vs PCOS before EA, ^∧^*P* < 0.05, ^∧∧^*P* < 0.01, ^∧∧∧^*P* < 0.001 vs unstimulated myotubes, data were analyzed by Student *t* test and presented as mean ± SEM. All investigated genes in this targeted approach are presented in Supplementary Table S1 (30). (j) Correlation of fold-changes of genes significantly modified in myotubes following EPS (*P* < 0.05) with genes changed in women by electroacupuncture. Spearman *r* = 0.46, *P* < 0.001. (k-n) Insulin stimulated glycogen incorporation in myotubes from healthy controls after gene silencing using siRNA for *AKAP13, HMOX1, MSX1, SRXN1* (n = 5-7). Data were analyzed by two-way ANOVA and presented as individual values, *P < 0.05 vs scramble control at baseline, and after 10 nM and 100 nM insulin stimulation.

We then applied a targeted approach to determine if a single bout of electroacupuncture altered gene expression in the same fashion in women with and without PCOS. Of the 44 genes previously identified by Illumina HumanHT-12 BeadChips array to be regulated by electroacupuncture in women with PCOS, 33 were technically validated by RT-qPCR using low-density array cards with the same samples (Table 2). Out of the 11 genes that were not validated, 9 showed decreased expression in the genome-wide gene expression array, with a smaller fold change compared to the upregulated genes. We found that expression of 21 of the 33 validated genes changed in the same direction in women with and without PCOS in response to electroacupuncture, with 20 being upregulated and 1 being downregulated (Fig. 6a-d, Table 2). Fifteen genes were regulated in a different direction in controls and in women with PCOS in response to electroacupuncture, including *PRINS*, *AKAP13*, *HMOX1*, *MSX1*, and *SRXN1* (Fig. 6e-i, Table 2).

**Table 2.** Targeted Gene Expression in Controls and Women With PCOS in Response to a Single Bout of Electroacupuncture (EA) and in Myotubes in Response to Electrical Pulse Stimulation (EPS)

Gene	Control after EA Fold change	*P*-value Control before vs after EA	PCOS after EA Fold change	*P*-value PCOS before vs after EA	Myotubes after EPS Fold change	*P*-value Unstimulated vs after EPS	Genes regulated in the same fashion in all groups
ATOH8	1,52 ± 0,11	**0,000**	1,56 ± 0,15	**0,000**	0,83 ± 0,19	**0,046**	
AKAP13	1,23 ± 0,08	**0,010**	0,79 ± 0,13	**0,013**	1,09 ± 0,17	0,151	
BCL3	1,73 ± 0,21	**0,007**	2,74 ± 0,30	**0,001**	1,32 ± 0,17	**0,006**	x
CCL2	8,33 ± 0,41	**0,000**	9,23 ± 0,58	**0,000**	2,56 ± 0,25	**0,008**	x
CCL8	5,06 ± 0,49	**0,003**	5,33 ± 0,44	**0,009**	0,67 ± 0,80	0,173	
CLDN5	0,84 ± 0,15	0,254	1,66 ± 0,23	**0,003**		ND	
CSDE1	1,07 ± 0,05	0,274	0,98 ± 0,11	0,946	0,97 ± 0,15	0,530	
CYR61	2,95 ± 0,23	**0,001**	7,50 ± 0,41	**0,001**	1,13 ± 0,19	0,138	
DENND2C	0,97 ± 0,14	0,696	0,75 ± 0,17	**0,015**	1,10 ± 0,17	0,304	
DNAJB1	1,81 ± 0,13	**0,000**	1,88 ± 0,28	**0,001**	1,67 ± 0,34	**0,034**	x
DYRK2	0,88 ± 0,11	0,136	1,02 ± 0,14	0,538	1,10 ± 0,16	0,313	
EHD1	1,20 ± 0,12	**0,007**	1,45 ± 0,09	**0,001**	1,04 ± 0,13	0,384	
ERF	1,39 ± 0,11	**0,002**	1,44 ± 0,21	**0,046**	1,15 ± 0,12	**0,000**	x
EXOC4	0,98 ± 0,10	0,794	0,92 ± 0,09	0,394	1,16 ± 0,12	**0,048**	
FAM110D	2,62 ± 0,41	**0,001**	2,52 ± 0,35	**0,002**		ND	
FAM43A	0,94 ± 0,15	0,497	1,57 ± 0,18	**0,010**	1,31 ± 0,55	0,103	
FBXO32	0,84 ± 0,15	**0,029**	0,87 ± 0,11	0,195	1,13 ± 0,23	**0,032**	
GADD45B	5,04 ± 0,49	**0,001**	8,78 ± 0,63	**0,001**	1,46 ± 0,23	**0,012**	x
HMOX1	1,11 ± 0,28	0,567	1,33 ± 0,28	**0,049**	3,58 ± 0,32	**0,000**	
ID2	1,64 ± 0,12	**0,000**	2,17 ± 0,20	**0,000**	1,07 ± 0,23	0,461	
IFIT3	1,11 ± 0,15	0,316	1,33 ± 0,30	0,359	1,26 ± 0,76	0,195	
KLF15	0,50 ± 0,24	**0,001**	0,51 ± 0,24	**0,000**	1,38 ± 0,20	**0,040**	
KLF2	1,48 ± 0,09	**0,008**	2,95 ± 0,17	**0,000**	1,12 ± 0,29	0,298	
LDLR	3,00 ± 0,22	**0,000**	3,12 ± 0,39	**0,004**	1,29 ± 0,21	**0,007**	x
MSX1	0,93 ± 0,15	0,756	2,65 ± 0,29	**0,008**	1,42 ± 0,24	**0,050**	
MT2A	1,36 ± 0,24	**0,001**	1,67 ± 0,21	**0,015**	1,62 ± 0,24	**0,022**	x
OSBPL9	0,93 ± 0,17	0,305	0,75 ± 0,16	**0,004**	1,14 ± 0,07	**0,001**	
PATJ	1,07 ± 0,06	0,404	0,82 ± 0,09	**0,048**	1,16 ± 0,13	**0,018**	
PDE4B	1,68 ± 0,10	**0,000**	2,08 ± 0,15	**0,000**	1,16 ± 0,31	0,299	
PDGFB	1,11 ± 0,11	0,201	1,30 ± 0,09	**0,004**	0,98 ± 0,45	0,822	
PLAU	1,46 ± 0,23	**0,030**	2,46 ± 0,30	**0,000**	0,78 ± 0,23	**0,003**	
PLEKHO2	1,70 ± 0,20	**0,007**	1,75 ± 0,25	0,076	1,19 ± 0,08	**0,001**	
PRINS	1,56 ± 0,12	**0,001**	0,59 ± 0,20	**0,004**	0,97 ± 0,58	0,768	
PTGER4	1,38 ± 0,16	**0,025**	1,94 ± 0,23	**0,002**	1,34 ± 0,15	**0,004**	x
RARA	1,22 ± 0,08	**0,048**	1,92 ± 0,18	**0,000**	1,01 ± 0,08	0,840	
RFX7	0,92 ± 0,06	0,053	0,92 ± 0,06	0,149	1,09 ± 0,13	0,150	
RYBP	1,26 ± 0,06	**0,001**	1,45 ± 0,11	**0,002**	1,26 ± 0,11	**0,001**	x
SLC2A3	4,43 ± 0,27	**0,000**	5,40 ± 0,39	**0,000**	1,29 ± 0,30	**0,031**	x
SORBS1	1,11 ± 0,10	0,277	0,83 ± 0,16	0,131	1,17 ± 0,22	**0,026**	
SRXN1	0,75 ± 0,08	**0,018**	1,64 ± 0,21	**0,008**	1,75 ± 0,15	**0,000**	
STAG3L3	1,08 ± 0,25	0,905	1,41 ± 0,27	0,459	0,98 ± 0,32	0,890	
STRIP2	1,06 ± 0,15	0,270	0,88 ± 0,15	0,486	1,06 ± 0,22	0,297	
TNFRSF1A	1,05 ± 0,10	0,635	1,23 ± 0,11	**0,038**	1,08 ± 0,09	0,097	
TNRC6B	1,29 ± 0,04	**0,000**	0,88 ± 0,06	0,144	1,16 ± 0,12	**0,048**	

Gene selection is presented in Supplementary Fig. S2. ND; not detected, data presented as mean ± SEM, analysed by Student *t* test (*P* < 0.05 in bold)

A method for providing electrical stimulation in vitro is electrical pulse stimulation (EPS) of differentiated human cultured myotubes. In response to EPS, 23 of the 44 investigated genes changed expression immediately post stimulation, with the majority of the genes increasing in expression (Table 2). We also compared the gene expression changes (using low-density array cards) in response to electroacupuncture in women without PCOS to the expression changes in EPS myotubes and found that 13 out of the 23 genes were upregulated (Table 2, Fig. 6a-d, g-i). Furthermore, 10 of the 13 genes were upregulated in all 3 paradigms investigated, i.e., in response to electroacupuncture in women with and without PCOS and in myotubes following EPS. There was a positive correlation for differentially expressed genes in skeletal muscle in women and genes that changed in response EPS in myotubes (*r*_*s*_* *= 0.46, Fig. 6j).

Finally, we investigated the effect of 4 differentially regulated genes on glucose incorporation into glycogen in human primary myotubes. siRNA-mediated silencing resulted in decreased gene expression of the target genes; *AKAP13*, *MSX1*, *HMOX1*, and *SRXN1* (Supplementary Fig. S2 (30)). Silencing of *AKAP13*, *MSX1*, and *HMOX1* decreased basal glucose incorporation into glycogen, while silencing of *MSX1* and *SRXN1* decreased insulin-stimulated glucose incorporation into glycogen (Fig. 6k-n).

## Discussion

Exercise positively remodels transcriptional and epigenetic profiles in skeletal muscle (45, 46), but whether electroacupuncture-induced contractions can induce similar benefits is unclear. Here we show for the first time that a single bout of low-frequency electroacupuncture alters DNA methylation and reverses PCOS-associated transcriptomic disturbances in skeletal muscle in a similar way as a single bout of exercise. Ninety-five percent of the genes altered by electroacupuncture were directed toward a healthier phenotype. Among these genes, over 50% exhibited changes in methylation levels in one or several CpG sites, with a majority of the methylation and expression changes going in opposite directions, suggesting that electroacupuncture has acute effects on the methylome, which in turn might affect the transcriptome.

Methylation of CpG sites affects the binding of transcription factors to DNA and thus alters gene expression (47). Using motif-enrichment analyses, we identified several transcription factors involved in the transcriptomic response to both electroacupuncture and exercise. Amongst them, the mRNA levels of *EGR1*, *EGR2*, and *ATF3* were increased 4- to 12-fold in response to electroacupuncture and in response to exercise. In addition to increasing their expression, electroacupuncture also induced methylation changes at CpG sites in these genes, suggesting that the induction of transcription in response to electroacupuncture partly involves methylation changes in transcription factor binding sites.

Kruppel like factor 4 (*KLF4*) and nuclear receptor subfamily 4 group A member 2 and 3 (*NR4A2, NR4A3*) are well-known exercise-responsive genes involved in muscle cell proliferation (48), and these genes were upregulated in response to electroacupuncture. *KLF4* regulates early insulin signaling (49) and is together with one of the most upregulated genes in response to electroacupuncture*, CCL2*, involved in the extracellular-signal-regulated kinase (ERK) 1 and ERK2 cascade. Activation of ERK signaling pathways is in line with increased ERK phosphorylation in rat skeletal muscle in response to electroacupuncture and exercise (50). Further, several CpG sites annotated to *KLF4* and *NR4A2* showed decreased methylation, which correlated with increased gene expression. Of note, the expression of NR4A3, which is associated with improved oxidative metabolism and muscle endurance in mice (51), was increased 750% after electroacupuncture. This key transcriptional regulator is also increased by acute exercise in healthy controls and type 2 diabetics, and this increase might be mediated by adenosine monophosphate-activated protein kinase (AMPK) activation (42, 48). Interestingly, the expression of *NR4A3* was also increased 10-fold after low-frequency electrical stimulation in paralyzed muscles (52). Collectively, these results suggest that it is the contractile activity, regardless of stimuli, i.e., exercise or electrical stimulation, that drives *NR4A3* expression and skeletal muscle adaptations.

The expression of dual-specificity tyrosine phosphorylation-regulated kinase 2 (*DYRK2*) correlates with the glucose infusion rate and is part of the same enriched signaling pathway as insulin receptor substrate 1 (*IRS1*) and sorbin and SH3 domain containing 1 (*SORBS1)*, both of which are involved in glycogen and glucose metabolism. All 3 genes were downregulated in response to electroacupuncture, further supporting the hypothesis that improved glucose homeostasis by electroacupuncture is mediated, at least in part, by downregulation of insulin signaling pathways. We hypothesize that the acute effect of electroacupuncture is insulin-independent but with long-term insulin-sensitizing effects. A similar tendency with unaltered insulin levels but improved glucose homeostasis, was seen in a human study using transcutaneous electrical stimulation with electrodes placed on the skin instead of needles inserted into the muscle, indicating a long-term insulin-sensitizing effect (53). Interestingly, electroacupuncture, but not exercise, stimulated signaling pathways involved in the regulation of glucose metabolism and glycogen biosynthesis, while signaling pathways involved in muscle tissue development and cellular responses to nutrient levels were upregulated in response to both exercise and electroacupuncture.

The majority of the technically validated genes in the targeted approach, using low-density array cards, were regulated in a similar manner in response to electroacupuncture in women with and without PCOS. Ten out of 21 genes were regulated in the same direction in EPS-treated myotubes in vitro, suggesting shared signaling pathways in these models, and supporting that EPS-myotubes is a valid model. Chemokine ligand 2 (*CCL2/ MCP1*), growth arrest and DNA damage-inducible beta (*GADD45B*), glucose transporter 3 (*SLC2A3*) and low-density lipoprotein receptor (*LDLR*) were the genes with the largest increases in expression in response to electroacupuncture, and they were also upregulated in EPS-treated human myotubes. CCL2, CCL8 and interleukin-6 are well-studied pro-inflammatory cytokines that are chronically elevated in obesity but they have also be identified as contraction-regulated myokines that have beneficial effects after exercise when levels increase acutely (54, 55). The increase after exercise is not necessarily due to muscle damage and inflammation, but is rather mediated by anti-inflammatory responses and metabolic adaptations in both healthy controls and women with PCOS (56, 57). *CCL2* and *LDLR* expression was upregulated in response to electroacupuncture and gene ontology analysis identified an upregulation of signaling pathways that negatively regulate responses to external stimuli and immune system processes, further supporting anti-inflammatory actions. The function of GLUT3 (*SLC2A3*) in human skeletal muscle is far from clear, but GLUT3 present in muscle might derive from myocytes and/or neural or vascular elements (58), and the available data imply that GLUT3 might be of importance for the glucose supply in regenerating adult muscle fibers post exercise (59).

In order to investigate the differential response to electroacupuncture in women with and without PCOS, we focused on genes with high mRNA expression in primary myotubes and that were shown to be differentially regulated in skeletal muscle from controls compared to skeletal muscle from PCOS. A-kinase anchoring protein 13 (*AKAP13*) was increased by electroacupuncture in controls but decreased in women with PCOS. Heme oxygenase 1 (*HMOX1*), Msh homeobox 1 *(MSX1)*, and sulfiredoxin-1 (*SRXN1*) had lower expression in skeletal muscle from women with PCOS at baseline, and the expression was increased after a single bout of electroacupuncture in women with PCOS but not in controls. siRNA-mediated silencing of *AKAP13*, *HMOX1*, or *MSX1* in primary myotubes resulted in decreased glucose incorporation into glycogen at baseline. Silencing of *MSX1* or *SRXN1* also resulted in decreased insulin-stimulated glucose incorporation into glycogen, supporting a role for these genes in glucose homeostasis. Interestingly, a decreased level of *MSX1* in human and mouse endometrial tissue is linked to infertility (60, 61), while *SRXN1* contributes to oxidative stress resistance (62). Our data suggest a possible novel role for these genes in the regulation of glucose metabolism.

Although insulin sensitivity is inhibited in obese and insulin-resistant individuals, insulin sensitivity improves with contractile activity (63, 64). Acutely exercise-stimulated glucose uptake by muscle occurs independently of insulin signaling, while the long-term effects of exercise include insulin sensitization for up to 48 hours (65). In vitro, primary myotubes cultured from obese, insulin-resistant donors exhibit impaired response to EPS compared to myotubes from lean controls (66), suggesting that despite defects in skeletal muscle cells associated with metabolic diseases, exercise-responsive molecules can be activated with contraction, but at a lower level compared to controls. The mechanisms behind electroacupuncture-stimulated glucose uptake in humans are less well characterized, but likely involve a combination of acute and long-term metabolic changes (20, 26). We previously found a similar increase in the glucose infusion rate in response to electroacupuncture in overweight and obese women with impaired insulin action compared to controls (22), suggesting that electroacupuncture regulates insulin sensitivity independently of the metabolic status of the individuals. Both exercise and electroacupuncture lead to increased GLUT3 and GLUT4 expression and the activation of glucose transport, which perhaps explains why insulin-resistant individuals nonetheless increase glucose uptake in response to an acute bout of exercise or electroacupuncture (20, 22, 67). Our data and the current literature therefore suggest that electroacupuncture and exercise act through partially similar signaling pathways to induce glucose uptake.

Our findings provide evidence that electroacupuncture normalizes gene expression in skeletal muscle in a manner similar to acute exercise. These results further highlight electroacupuncture as a therapeutic means to ameliorate metabolic disturbances in women with PCOS, especially in individuals who have difficulties performing exercise.

## Abbreviations

BMI: body mass index
EPS: electrical pulse stimulation
FDR: false discovery rate
PCOS: polycystic ovary syndrome

## Additional Information

***Disclosure Summary:*** The funders had no role in the study design, data collection and analysis, decision to publish, or preparation of the manuscript. Each author declares that (s)he has no relevant or material financial interests that relate to the research described in this paper.

***Data Availability:*** In addition to the referenced data available through Dryad (30), all raw global gene and DNA methylation analyses are available. A detailed description of the case-control studies including global gene expression analyses in subcutaneous adipose tissue and skeletal muscle has previously been published (13, 14, 23). Any other data described in the analysis of this study are available from the authors upon a reasonable request.
